# Screening Yield of Inherited Bleeding Disorder Questionnaires in Women With Chronic Heavy Menstrual Bleeding (HMB): A Cross-Sectional Study

**DOI:** 10.7759/cureus.91994

**Published:** 2025-09-10

**Authors:** Anum Faiz, Madina Eltayeb Dawelbait Radwan, Safa Iqbal, Sagar Vinayak, Deepti Gowda, Kaushalendra Mani Tripathi, Ghulam M Mahmood, Hamaida Akbar, Mubashra Kanwal, Ankita Sunil, Racha Al Niazi, Aurooba Naeem

**Affiliations:** 1 Cardiology Medicine, Jinnah Hospital Lahore, Lahore, PAK; 2 Obstetrics and Gynecology, Aljabal Primary Health Care Center, Jazan, SAU; 3 Neurosurgery, Shifa International Hospitals Limited, Islamabad, PAK; 4 Pathology, Pakistan Atomic Energy General Hospital, Islamabad, PAK; 5 Internal Medicine, American University of Barbados School of Medicine, Bridgetown, BRB; 6 Medicine, Hull University Teaching Hospitals NHS Trust, Hull, GBR; 7 Internal Medicine, White River Medical Center, Batesville, USA; 8 Internal Medicine, King Edward Medical University, Lahore, PAK; 9 Internal Medicine, Fatima Memorial Hospital, Lahore, PAK; 10 Internal Medicine, Mayo Hospital, Lahore, PAK; 11 Internal Medicine, Thumbay University Hospital, Ajman, ARE; 12 Orthodontics, Dubai Health, Dubai, ARE; 13 Medicine, Shifa International Hospitals Limited, Islamabad, PAK

**Keywords:** diagnostic yield, heavy menstrual bleeding, inherited bleeding disorders, menstrual bleeding questionnaire, pakistan

## Abstract

Background

Chronic disease, heavy menstrual bleeding (HMB), is among the common conditions that may negatively impact the overall health and quality of life of women. HMB is commonly associated with inherited bleeding disorders (IBDs); nonetheless, it remains underdiagnosed in Pakistan. Early detection may result in a more successful curative regimen and the prevention of unnecessary interventions, made possible by screening. This research aimed to investigate the diagnostic yield of an IBD questionnaire screening in women with persistent HMB.

Methods

The research study used a cross-sectional design at the Outpatient Gynecology Department of Shifa International Hospitals Limited, Islamabad, Pakistan, between October 2024 and June 2025. Convenience sampling was used to recruit 350 women with self-reported chronic HMB, confirmed by structured history, who were included. A structured questionnaire containing demographic information, as well as sections from the International Society on Thrombosis and Haemostasis Bleeding Assessment Tool (ISTH-BAT) and the Menstrual Bleeding Questionnaire (MBQ), was used to collect the data. Descriptive statistics, correlation, independent t-tests, ANOVA, chi-square tests, and multiple regression analyses were performed using IBM SPSS Statistics for Windows, Version 26 (Released 2019; IBM Corp., Armonk, NY, USA).

Results

Of the total, 229 (65%) of the participants had a history of a diagnosis of an IBD, and 208 (59%) had a family history of a bleeding disorder. Women with a family history and women with IBDs had significantly higher mean ISTH-BAT and MBQ scores (p < 0.001). There were positive associations between ISTH-BAT scores and MBQ (r = 0.312, p < 0.001), indicating that higher ISTH-BAT scores were associated with a greater perception of menstrual burden. Among the IBDs, von Willebrand disease had the highest scores. Regression analysis showed that ISTH-BAT scores (B = 0.165, 95% CI: 0.076-0.254, p < 0.001), age (B = 0.328, 95% CI: 0.052-0.604, p = 0.020), family history (B = 0.842, 95% CI: 0.272-1.412, p = 0.004), previous diagnosis (B = 0.612, 95% CI: 0.109-1.115, p = 0.017), and the presence of bleeding symptoms (B = 0.533, 95% CI: 0.130-0.936, p = 0.010) were significant predictors of MBQ scores (R² = 0.362).

Conclusions

Screening via questionnaire was effective in detecting women at risk of IBDs among those with chronic HMB. Standardized bleeding assessment in gynecological practice may enable many women to be diagnosed earlier, undergo effective treatment, and avoid anemia and unnecessary surgical procedures.

## Introduction

Heavy menstrual bleeding (HMB), defined as a volume of blood loss (VBL) of 80 mL per cycle, may be attributed to uterine pathologies, ovulatory abnormalities, and - in up to 20% of cases - to inherited bleeding disorders (IBDs) [1]. In Pakistan, a hospital-based study reported that 37.9% of women of reproductive age experienced HMB, which was associated with anemia, fatigue, and reduced quality of life [2]. Among women experiencing HMB, the most distressing aspects were pain, heaviness, and the impact of symptoms on daily life. The bleeding was deemed to be heavy, yet this was subjective, reflecting the necessity of individualized approaches to clinical support and symptom management [3].

Both adolescents and reproductive-aged women experience HMB, which may cause anemia and significantly affect quality of life. Early detection and treatment interventions - such as hormonal methods, including the use of a levonorgestrel intrauterine device - are crucial for improving outcomes and treating resultant iron deficiency [4,5]. Various medical therapies, including oral progestogens and intrauterine systems, are commonly prescribed for HMB [6]. However, women with undiagnosed IBDs may not respond adequately to these treatments, underscoring the importance of timely identification.

Women with IBDs encounter a wide range of issues, the most common one being HMB. The condition significantly restricts their quality of life, increases the risk of iron-deficiency anemia, and, in some cases, is compounded by stigma and taboos - causing many women to avoid seeking medical advice [7,8]. Moreover, women with IBDs face numerous diagnostic uncertainties and are burdened with harsh symptoms that interfere with their identities and daily lives [9].

Increased awareness and diagnosis of inherited bleeding conditions are considered crucial to managing these diseases, particularly in women with hemophilia. These women often experience excessive menstrual bleeding that goes unrecognized. Bleeding disorders such as von Willebrand disease and factor XI deficiency should be diagnosed early to avoid invasive procedures and improve management [10,11].

Rationale

Chronic HMB is one of the most common problems faced by a considerable number of women across the globe, causing physical and psychological burdens. Although it is a well-known fact that acquired conditions, like the presence of fibroids and hormonal imbalances, contribute to HMB, IBDs are an underreported phenomenon in most areas, including Pakistan. Hereditary disorders, such as von Willebrand disease, platelet dysfunction, and other coagulation anomalies, may lead to significant menstrual bleeding, but they are not well-known or well-screened. In Pakistan, routine screening for IBDs in women with chronic HMB is limited. While our study was conducted in a major urban center, such diagnostic practices are even less common in secondary and rural healthcare settings, increasing the risk of misdiagnosis or delayed treatment.

The high incidence of IBDs among women with chronic HMB is a largely unfamiliar field in Pakistan. There is a need to investigate the role of these conditions in the development of HMB within local populations and to assess the diagnostic yield of screening for these conditions. The possibility of potential improvement in management and outcomes when individuals are recognized in time allows the burden of these disorders in women with chronic HMB to be leveraged to influence clinical practices, reduce unnecessary interventions, and provide the basis for population health policy. The study aims to evaluate the screening yield of questionnaire-based identification of IBDs in women with chronic HMB in Pakistan, and to provide evidence to inform local clinical practice and policy. This study evaluates the potential of questionnaire-based screening to identify women at risk for IBDs. Confirmatory diagnostic tests were not performed.

Primary objectives

The study aimed to investigate whether a questionnaire-based approach to screening for IBDs has utility in identifying women at risk in the setting of chronic HMB in Pakistan.

Secondary objectives

The secondary objectives of this study are to assess the proportion of women with chronic HMB who screen positive for specific heritable bleeding disorders (e.g., von Willebrand disease, platelet functional disorders, clotting factor deficiencies) using a questionnaire; to explore the relationship between demographic factors (e.g., age and family history) and the likelihood of screening positive for inherited bleeding conditions in this group of patients; and to assess the severity of menstrual bleeding as compared to the presence and type of IBD, as determined by the questionnaire.

## Materials and methods

The research study used a cross-sectional design to investigate the diagnostic yield of screening Pakistani women with chronic HMB for IBDs. Participants were recruited through the Gynecology Outpatient Department of Shifa International Hospitals Limited, Islamabad, Pakistan, and provided written or oral informed consent to ensure their ethical participation.

Information was obtained using a structured questionnaire that aimed to determine whether individuals exhibit symptoms of IBDs and whether these symptoms are related to chronic HMB. Women were contacted during regular gynecological follow-ups conducted by trained research assistants, who informed them of the study's purpose and obtained informed consent. The participants had plenty of time to discuss the study details and clarify any questions, after which they were expected to agree to participate. Only women who had given written or oral consent to participate in the study were recorded. The participants were provided with a questionnaire and asked to complete it separately, according to their level of literacy, during an interview with a research assistant. This strategy ensured clarity and respect during communication, taking into account cultural differences and the healthcare setting in Pakistan.

Sampling strategy and population size

The sample size was calculated assuming an infinite population, with an estimated prevalence (p) of 0.5 in the absence of local data, a 95% confidence level (Z = 1.96), and a margin of error of 0.05 [12].

The women were recruited through a convenience sample attending gynecology outpatient clinics in hospitals within Islamabad and Rawalpindi. Of the 380 women approached, 30 were ineligible or declined participation. The remaining 350 participants consented, completed the questionnaire, and were included in the analysis (92.1% of 380). Although convenience sampling offers the advantage of efficiency in sample selection and resource allocation, it is also a potential source of selection bias, which may restrict the generalizability of the results to the broader population beyond the clinics chosen in Islamabad and Rawalpindi.

Inclusion and exclusion criteria

The participants were considered to have chronic HMB and experienced bleeding for more than seven days, passing large clots, or changing pads/tampons regularly. They were aged 18 years and older, with no previous diagnosis of an IBD. The participants were required to attend gynecology outpatient clinics in hospitals within the cities of Islamabad and Rawalpindi and agree to give informed consent. Women excluded included those with a known IBD, a known acquired bleeding disorder, a history of hysterectomy or other major surgeries that might interfere with menstruation, pregnancy or less than six months post-delivery, severe medical conditions that might influence bleeding, or those unwilling or unable to consent.

Data collection tools

A structured questionnaire was designed to collect pertinent and detailed data in the context of this study, which included three primary items: demographic details, screening for inherited bleeding conditions, and evaluation of excessive menstrual bleeding (Table 11, see Appendix). All tools applied were in their original language, which is English, and the participants were able to provide the correct answers without requiring language or cultural adjustments.

Demographic information

The first part of the questionnaire collected data on demographics and medical history to facilitate subgroup analysis and explore the potential correlation between these factors and the occurrence of hereditary bleeding disorders. Variables included age, marital status, level of education, and occupation. Clinical data included menstrual histories, presence or absence of comorbidities, family history of bleeding problems, and history of medical treatment for HMB.

International Society on Thrombosis and Haemostasis Bleeding Assessment Tool (ISTH-BAT)

The ISTH-BAT, developed initially by Rodeghiero et al. in 2010, is a standardized instrument that assesses bleeding symptoms across 14 categories (e.g., epistaxis, cutaneous bleeding, minor wounds, menorrhagia, postsurgical bleeding). Each category is scored according to the worst lifetime episode, using category-specific criteria that reflect the clinical impact and interventions required (e.g., medical consultation, transfusion). The total bleeding score is obtained by summing across categories. For the present study, a modified version of the ISTH-BAT was administered, comprising only four categories - epistaxis, cutaneous bleeding, minor wounds, and menorrhagia - selected for their direct relevance to women with chronic HMB (Table 12, see Appendix). Other sections (e.g., gastrointestinal, urinary, postsurgical bleeding) were omitted to minimize participant burden and because they were less pertinent to the study objectives. The four-category scores were analyzed as a subset of ISTH-BAT items, rather than as a full ISTH-BAT score, and no published BAT cutoffs were applied. Permission to use the tool was obtained from the original authors [13].

Menstrual Bleeding Questionnaire (MBQ)

The third component evaluated the impact and severity of HMB using the MBQ (Table 13, see Appendix), which was established in 2015 by Matteson et al. [14]. This is a patient-reported measure assessing the intensity and duration of menstrual bleeding, the presence of clots, the frequency of pad/tampon changes, the experience of pain associated with menstrual bleeding, and the impact of HMB on day-to-day activity levels and overall quality of life. The MBQ consists of 20 questions, divided into four domains: heaviness, irregularity of bleeding, pain, and quality of life. Each item is rated on a 0-4 scale, where higher scores are assigned to more severe or impactful symptoms. The sum of all items provides a general evaluation of the menstrual bleeding and its effects. The instrument exhibits excellent reliability, with Cronbach's alpha ranging from 0.87 to 0.94 [14]. The MBQ is widely used in various research studies and was employed in this study with the consent of the original authors.

Procedure

Participants were recruited using written or oral informed consent to participate in the study sample, from the Gynecology Department of Shifa International Hospitals Limited in Islamabad. The data were collected over a period of five months, from October 2024 to June 2025. After making a routine clinic visit, trained research assistants approached women who presented with chronic HMB. The structured questionnaire was either self-administered or administered by interview, depending on the participant's literacy level and their preference. All data were anonymized to ensure confidentiality and preserve the participants' identities. The research was carried out in an ethical, respectful, and inclusive manner, allowing the participation of individuals from diverse demographic, cultural, and socioeconomic backgrounds. This approach helped capture a broad range of demographic and clinical profiles among women with HMB in Islamabad, while acknowledging the inherent limitations of convenience sampling.

Statistical analysis

The data were analyzed with IBM SPSS Statistics for Windows, Version 26 (Released 2019; IBM Corp., Armonk, NY, USA). The demographic and clinical characteristics of the 350 participants were characterized using descriptive statistics, including frequencies and percentages. Kolmogorov-Smirnov and Shapiro-Wilk tests were conducted to evaluate the normality of the main variables, including ISTH-BAT and MBQ scores. Pearson correlation analysis was used to study the correlation between the ISTH-BAT and MBQ scores. Independent sample t-tests were performed to compare the mean scores based on family history of bleeding disorders or the existence of diagnosed bleeding disorders. A one-way ANOVA was conducted to compare the mean scores across various age groups and IBDs. Multiple linear regression was used to determine the predictors of menstrual bleeding severity (MBQ scores), namely ISTH-BAT scores, age, family history, diagnosis status, type of bleeding disorder, and the existence of bleeding-related symptoms. Chi-square tests were used to analyze the relationship between categorical variables - such as age groups, diagnosis status, and type of bleeding disorder - and treatment outcomes. All statistical tests were two-sided, and significance was set at p < 0.05.

Ethical considerations

The research process adhered to internationally accepted ethical principles for conducting a research study involving human participants. The research procedure was granted a permit by the Institutional Review Board (IRB) of Shifa International Hospitals, Islamabad, with authorization number 0309-24. Therefore, the study complies with ethical standards, including respect for persons, beneficence, and confidentiality. All participants provided informed consent, and their participation was entirely voluntary. Confidentiality and privacy were maintained, and all data gathered were anonymized. To maximize data integrity, unusable or incomplete records were excluded from the final analysis.

## Results

Table 1 shows the demographics and clinical features of the 350 participants. The largest subgroup of participants (N = 120, 34%) were between 26 and 35 years old, followed by those between 36 and 45 years of age (N = 110, 31%), 18 and 25 years of age (N = 90, 26%), and 46 and 55 years of age (N = 30, 9%). As far as marital status is concerned, 147 (42%) were single, 142 (41%) married, 51 (15%) divorced, and 10 (3%) were widowed. Educational levels were mainly at the primary school (N = 119, 34%) and secondary school levels (N = 118, 34%), with a smaller proportion having no education (N = 45, 13%), college/university education (N = 55, 16%), or postgraduate/doctoral education (N = 13, 4%). Occupationally, 123 respondents (35%) were employed, 122 (35%) were homemakers, 55 (16%) were retired, and 50 (14%) were students. A considerable proportion of participants reported a family history of a bleeding disorder (N = 208, 59%) and a previous or suspected diagnosis based on self-report or screening findings (N = 229, 65%). Among these, the most commonly reported were von Willebrand disease (N = 137, 39%) and hemophilia (N = 126, 36%). These figures reflect self-reported or screening-positive cases rather than confirmed diagnoses and should be interpreted with caution. The most common manifestations included frequent nosebleeds (N = 115, 33%) and long-lasting bleeding following cuts (N = 89, 25%). The majority of participants (N = 225, 64%) had sought treatment for excessive menstrual bleeding, and a significant proportion (N = 193, 55%) engaged in regular physical exercise. Just over half of the participants had a history of smoking (N = 197, 56%).

**Table 1:** Demographic Characteristics of Participants (N = 350) This table presents the demographic and clinical features of women with chronic heavy menstrual bleeding (HMB), including age, marital status, education, occupation, family history of bleeding disorders, prior diagnosis of a bleeding disorder, symptoms, treatment history, smoking status, and physical activity. Values are given as frequency (N = 350) and percentage (%). No statistical comparisons were performed in this descriptive table.

Variable	f (N)	%
Age		
18-25 years	90	26
26-35 years	120	34
36-45 years	110	31
46-55 years	30	9
Marital status		
Single	147	42
Married	142	41
Divorced	51	15
Widowed	10	3
Educational level		
No formal education	45	13
Primary school	119	34
Secondary school	118	34
College/university	55	16
Postgraduate/doctoral	13	4
Occupation		
Student	50	14
Employed	123	35
Homemaker	122	35
Retired	55	16
Do you have a family history of bleeding disorders (e.g., hemophilia, von Willebrand disease)?		
No	142	41
Yes	208	59
Have you ever been diagnosed with a bleeding disorder?		
No	121	35
Yes	229	65
If yes, which bleeding disorder were you diagnosed with?		
von Willebrand disease	137	39
Hemophilia	126	36
Platelet disorders	75	21
Other	12	3
Do you experience any of the following symptoms?		
Easy bruising	53	15
Frequent nosebleeds	115	33
Prolonged bleeding after cuts	89	25
Bleeding gums	61	17
Joint or muscle bleeding	27	8
None of the above	5	1
Have you received treatment for heavy menstrual bleeding (e.g., antifibrinolytic agents, hormonal therapy)?		
No	125	36
Yes	225	64
Do you smoke?		
No	153	44
Yes	197	56
Do you engage in regular physical exercise?		
Yes, regularly	193	55
Occasionally	128	37
No	29	8

Table 2 presents the normality outcomes of ISTH-BAT and MBQ scores. Kolmogorov-Smirnov and Shapiro-Wilk tests revealed that the data were normally distributed for both ISTH-BAT (Kolmogorov-Smirnov: statistic = 0.032, df = 350, p = 0.200; Shapiro-Wilk: statistic = 0.997, df = 350, p = 0.485) and MBQ (Kolmogorov-Smirnov: statistic = 0.028, df = 350, p = 0.200; Shapiro-Wilk: statistic = 0.996, df = 350, p = 0.274). The assumption of normality was met, as all p-values were greater than 0.05, supporting the use of parametric tests in subsequent analyses.

**Table 2:** Tests of Normality for ISTH-BAT and MBQ Values are presented as test statistic (df = 350), with corresponding p-values. A p-value > 0.05 was considered statistically significant, indicating normal distribution (N = 350). Kolmogorov-Smirnov and Shapiro-Wilk tests were applied to examine the normality of ISTH-BAT and MBQ scores. Both tests showed p > 0.05, indicating normal distribution of the data and supporting the use of parametric analyses in subsequent tests. ISTH-BAT and MBQ were used with permission. ISTH-BAT, International Society on Thrombosis and Haemostasis Bleeding Assessment Tool; MBQ, Menstrual Bleeding Questionnaire

Variables	Kolmogorov-Smirnov			Shapiro-Wilk		
	Statistic	df	p	Statistic	df	p
ISTH-BAT	0.032	350	0.200	0.997	350	0.485
MBQ	0.028	350	0.200	0.996	350	0.274

Table 3 illustrates that ISTH-BAT scores show a moderate positive correlation with MBQ scores (r = 0.312, p < 0.001). This suggests that higher ISTH-BAT scores are associated with higher MBQ scores, indicating more severe menstrual bleeding alongside a greater overall bleeding tendency. The correlation is considered statistically significant at the 0.01 level, reflecting a relationship between overall bleeding symptoms and the severity of menstrual bleeding among the participants.

**Table 3:** Pearson Correlations Between ISTH-BAT and MBQ Scores (N = 350) Values represent Pearson correlation coefficients (r) between continuous variables; p < 0.01 (two-tailed) was considered statistically significant and is denoted with double asterisks (**). Pearson correlation analysis was conducted to examine the relationship between ISTH-BAT and MBQ scores. A significant positive correlation (r = 0.312, p < 0.001) was found, suggesting that higher bleeding tendency scores were associated with a greater menstrual burden. ISTH-BAT and MBQ were used with permission. ISTH-BAT, International Society on Thrombosis and Haemostasis Bleeding Assessment Tool; MBQ, Menstrual Bleeding Questionnaire

Variable	1	2	p
ISTH-BAT	-	0.312^**^	<0.001^**^
MBQ	0.312^**^	-	<0.001^**^

Table 4 indicates that participants who had a family history of bleeding disorders exhibited significantly higher scores on both the ISTH-BAT (M = 69.18, SD = 7.01) and MBQ (M = 50.35, SD = 4.77), compared to participants who did not report a family history (ISTH-BAT: M = 65.42, SD = 7.81; MBQ: M = 48.10, SD = 5.23). All these differences were statistically significant (ISTH-BAT: t(348) = 4.87, p < 0.001, d = 0.51; MBQ: t(348) = 4.10, p < 0.001, d = 0.45), with a moderate effect size observed. This implies that a positive family history of bleeding disorders is associated with increased overall bleeding predisposition and more severe menstrual bleeding among the participants.

**Table 4:** Independent Samples t-test Comparing Participants With and Without a Family History of Bleeding Disorders on ISTH-BAT and MBQ Scores (N = 350) Values are presented as mean ± standard deviation. Independent samples t-tests were conducted to compare participants with and without a family history of bleeding disorders. Group sizes are shown as N (%). Reported statistics include p-values, t-values, 95% confidence intervals (CI), and effect sizes (Cohen’s d). A p-value < 0.01** was considered statistically significant (N = 350). Independent t-tests compared mean ISTH-BAT and MBQ scores between women reporting a family history of bleeding disorders and those without. Women with a positive family history had significantly higher scores on both ISTH-BAT and MBQ (p < 0.001), indicating increased bleeding tendency and menstrual severity. ISTH-BAT and MBQ were used with permission. ISTH-BAT, International Society on Thrombosis and Haemostasis Bleeding Assessment Tool; MBQ, Menstrual Bleeding Questionnaire

Variable	No (N = 142; 41%), M ± SD	Yes (N = 208; 59%), M ± SD	t	p	95% CI Lower Limit (LL)	95% CI Upper Limit (UL)	Cohen’s d
ISTH-BAT	65.42 ± 7.812	69.18 ± 7.005	-4.873	<0.001^**^	-5.28	-2.24	-0.51
MBQ	48.10 ± 5.230	50.35 ± 4.772	-4.099	<0.001^**^	-3.33	-1.17	-0.45

Table 5 reveals that participants who self-reported a previous or suspected bleeding disorder based on family history or screening results had significantly higher scores on the ISTH-BAT (M = 68.72, SD = 8.01) and the MBQ (M = 50.52, SD = 4.78), compared with participants without a self-reported or suspected bleeding disorder (ISTH-BAT: M = 65.42, SD = 7.21; MBQ: M = 48.35, SD = 5.42). All these differences were statistically significant (ISTH-BAT: t = 3.921, p < 0.001, Cohen’s d = 0.43; MBQ: t = 3.760, p < 0.001, Cohen’s d = 0.43), with a moderate effect size. This indicates that the diagnosis of bleeding disorders is associated with a greater overall bleeding tendency and more significant menstrual bleeding among the participants.

**Table 5:** Independent Samples t-test Comparing ISTH-BAT and MBQ Scores by Bleeding Disorder Diagnosis (N = 350) Values are presented as mean ± standard deviation. Independent samples t-tests were conducted to compare participants with and without bleeding disorders. Group sizes are shown as N (%). Reported statistics include p-values, t-values, 95% confidence intervals (CI), and effect sizes (Cohen’s d). A p-value < 0.01** was considered statistically significant (N = 350). Mean ISTH-BAT and MBQ scores were compared between women with and without a diagnosed bleeding disorder. Scores were significantly higher among diagnosed women (p < 0.001), reflecting greater overall bleeding tendency and menstrual burden. ISTH-BAT and MBQ were used with permission. ISTH-BAT, International Society on Thrombosis and Haemostasis Bleeding Assessment Tool; MBQ, Menstrual Bleeding Questionnaire

Variable	No (N = 121; 35%), M ± SD	Yes (N = 229; 65%), M ± SD	t	p	95% CI Lower Limit (LL)	95% CI Upper Limit (UL)	Cohen’s d
ISTH-BAT	65.42 ± 7.210	68.72 ± 8.005	3.921	<0.001^**^	1.64	4.96	0.426
MBQ	48.35 ± 5.420	50.52 ± 4.780	3.760	<0.001^**^	1.03	3.32	0.433

Table 6 presents the one-way ANOVA outcomes comparing ISTH-BAT and MBQ scores across age categories. Both ISTH-BAT and MBQ scores differed significantly across age groups, with the highest scores observed in the 46-55 years age range (ISTH-BAT: M = 72.50, SD = 7.20; MBQ: M = 53.20, SD = 4.40). The age-group variance was statistically significant (ISTH-BAT: F(3, 346) = 7.02, p < 0.001, η² = 0.06; MBQ: F(3, 346) = 17.34, p < 0.001, η² = 0.13), suggesting a small to moderate contribution of age to the overall bleeding tendency and the severity of menstrual bleeding. Older participants had higher reported bleeding symptoms and more severe menstrual bleeding, as indicated by their ISTH-BAT and MBQ scores.

**Table 6:** One-Way ANOVA Results for ISTH-BAT and MBQ Scores by Age Group (N = 350) Data are presented as mean ± standard deviation (M ± SD). Group sizes are shown as N (%). One-way ANOVA was conducted to examine the effect. A p-value < 0.01** was considered statistically significant. η² represents partial eta-squared effect size. One-way ANOVA was used to compare mean ISTH-BAT and MBQ scores across four age groups (18-25, 26-35, 36-45, and 46-55 years). Significant differences were found (p < 0.001), with older women scoring higher on both measures, indicating an increase in bleeding severity and menstrual burden with age. Partial eta-squared (η²) values indicated small to moderate effect sizes. ISTH-BAT and MBQ were used with permission. ISTH-BAT, International Society on Thrombosis and Haemostasis Bleeding Assessment Tool; MBQ, Menstrual Bleeding Questionnaire

Age	18-25 years (N = 90; 26%), M ± SD	26-35 years (N = 120; 34%), M ± SD	36-45 years (N = 110; 31%), M ± SD	45-55 years (N = 30; 9%), M ± SD	p	F (3, 346)	η2
ISTH-BAT	65.20 ± 6.80	68.40 ± 7.95	70.10 ± 8.25	72.50 ± 7.20	<0.001^**^	7.02	0.06
MBQ	47.80 ± 4.85	49.30 ± 4.75	51.10 ± 5.10	53.20 ± 4.40	<0.001^**^	17.34	0.13

Table 7 presents the results of a one-way ANOVA analysis on data regarding IBD types in ISTH-BAT and MBQ scores from 350 study participants. There was a significant mean group difference on both ISTH-BAT (F(3, 346) = 7.45, p < 0.001, η² = 0.061) and MBQ (F(3, 346) = 13.80, p < 0.001, η² = 0.107). Bleeding severity was highest in patients with von Willebrand disease (M = 70.60, SD = 8.40), and menstrual bleeding burden was also highest in patients with von Willebrand disease (M = 51.30, SD = 5.20), followed by hemophilia, platelet disorders, and other IBDs. The medium effect sizes indicate that bleeding disorder type was associated with differences in general bleeding symptoms as well as menstrual-related bleeding.

**Table 7:** One-way ANOVA Comparing Inherited Bleeding Disorder Types on ISTH-BAT and MBQ Scores (N = 350) Data are presented as mean ± standard deviation (M ± SD); group sizes are shown as N (%). One-way ANOVA was conducted to compare inherited bleeding disorder types on ISTH-BAT and MBQ scores. A p-value < 0.01** was considered statistically significant; η² represents the partial eta-squared effect size. ANOVA tested differences in ISTH-BAT and MBQ scores across von Willebrand disease, hemophilia, platelet disorders, and other rare disorders. von Willebrand disease had the highest mean scores on both scales, followed by hemophilia and platelet disorders (p < 0.001). Results confirm variation in bleeding and menstrual burden across disorder types. ISTH-BAT and MBQ were used with permission. ISTH-BAT, International Society on Thrombosis and Haemostasis Bleeding Assessment Tool; MBQ, Menstrual Bleeding Questionnaire

Inherited Bleeding Disorder Types	von Willebrand Disease (N = 137; 39%), M ± SD	Hemophilia (N = 126; 36%), M ± SD	Platelet Disorders (N = 75; 21%), M ± SD	Other (N = 12; 3%), M ± SD	p	F (3, 346)	η2
ISTH-BAT	70.60 ± 8.40	67.50 ± 8.10	64.20 ± 7.85	61.30 ± 7.10	<0.001^**^	7.45	0.061
MBQ	51.30 ± 5.20	49.60 ± 5.00	47.60 ± 4.90	45.20 ± 4.60	<0.001^**^	13.80	0.107

Table 8 indicates that several demographic and clinical factors contributed significantly to predicting MBQ scores, with a percentage variance of approximately 36.2% (R² = 0.362, adjusted R² = 0.348). More severe menstrual bleeding was significantly associated with higher ISTH-BAT scores (β = 0.215, p < 0.001), older age (β = 0.105, p = 0.020), positive family history of bleeding disorders (β = 0.142, p = 0.004), prior diagnosis of a bleeding disorder (β = 0.118, p = 0.017), type of bleeding disorder (β = 0.097, p = 0.030), and presence of bleeding-related symptoms (β = 0.121, p = 0.010). These results indicate that clinical characteristics and personal/family history were associated with menstrual bleeding severity in participants.

**Table 8:** Multiple Regression Analysis Predicting MBQ Scores From Demographic and Clinical Variables (N = 350) Multiple linear regression was conducted to identify predictors of MBQ scores. Values include unstandardized coefficients (B), 95% confidence intervals (CI), standard error (SE), standardized beta coefficients (β), and p-values. A p-value < 0.05* and p-value < 0.01** was considered statistically significant (N = 350). Multiple linear regression was conducted with MBQ scores as the outcome variable, and predictors including ISTH-BAT, age, family history, diagnosis status, disorder type, and bleeding symptoms. The model was significant (R² = 0.362, p < 0.001). Higher ISTH-BAT scores, older age, a positive family history, prior diagnosis, disorder type, and the presence of symptoms were all significant predictors of greater menstrual burden. MBQ used with permission. ISTH-BAT, International Society on Thrombosis and Haemostasis Bleeding Assessment Tool; MBQ, Menstrual Bleeding Questionnaire

Predictor	B	SE	β	t	p	95% CI Lower Limit (LL)	95% CI Upper Limit (UL)
Constant (MBQ)	29.842	2.480	-	12.030	<0.001^**^	24.965	34.719
ISTH-BAT	0.165	0.045	0.215	3.667	<0.001^**^	0.076	0.254
Age	0.328	0.140	0.105	2.343	0.020^*^	0.052	0.604
Family history of bleeding disorders	0.842	0.290	0.142	2.902	0.004^**^	0.272	1.412
Ever diagnosed with a bleeding disorder	0.612	0.255	0.118	2.400	0.017^*^	0.109	1.115
Type of bleeding disorder diagnosed	0.458	0.210	0.097	2.181	0.030^*^	0.045	0.871
Presence of bleeding-related symptoms	0.533	0.205	0.121	2.595	0.010^*^	0.130	0.936
Model summary	R	R^2^	Adjusted R^2^	SE of the estimate	-	-	-
	0.601	0.362	0.348	4.12	-	-	-

**Figure 1:**
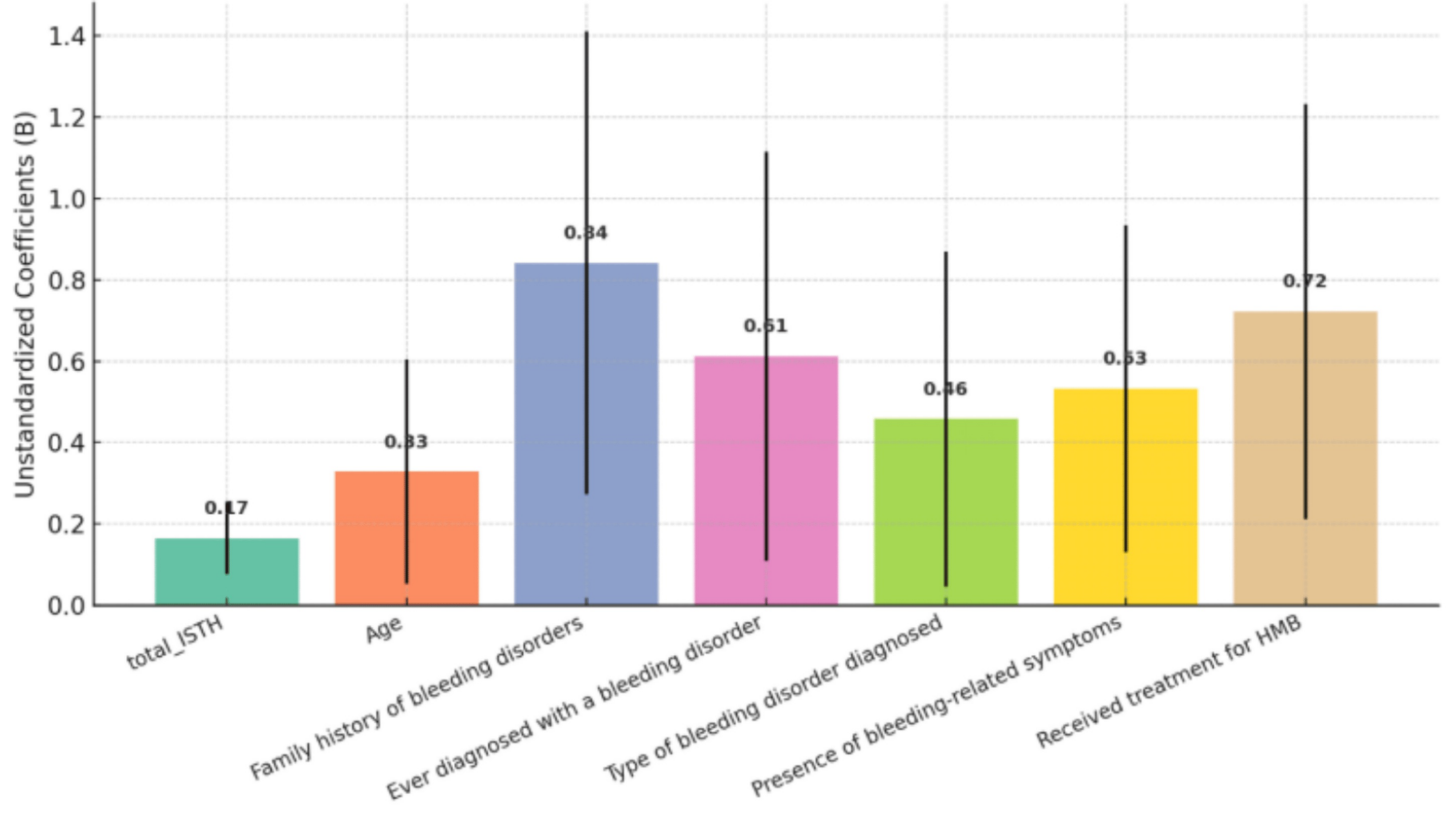
Histogram of Regression Coefficients With 95% Confidence Intervals for Predictors of MBQ Scores This figure illustrates unstandardized regression coefficients (B), with 95% confidence intervals, for predictors of MBQ scores from the multiple regression model. Variables with positive coefficients were associated with greater menstrual burden. MBQ used with permission. ISTH, International Society on Thrombosis and Haemostasis; MBQ, Menstrual Bleeding Questionnaire; HMB, Heavy Menstrual Bleeding

Figure 1 illustrates the regression coefficients (B) and 95% confidence intervals of the predictors associated with MBQ scores. The strongest associations with higher MBQ scores were observed for a history of ever being diagnosed with a bleeding disorder (B = 0.84), the type of bleeding disorder diagnosed (B = 0.61), and treatment received for HMB (B = 0.72). Bleeding-related event symptoms (B = 0.53) and age (B = 0.33) showed moderate associations, while a family history of bleeding conditions (B = 0.17) showed the weakest association. Confidence intervals were wide for the majority of predictors, suggesting that these estimates should be interpreted cautiously. Overall, a clinical history of bleeding disorders and treatment for HMB were most strongly associated with higher MBQ scores.

Table 9 shows a statistically significant relationship between the IBD and treatment outcome (χ²(3) = 8.869, p = 0.031, Cramer's V = 0.159). Participants with von Willebrand disease (99; 28%) and hemophilia (77; 22%) were most likely to have been treated, compared with those with platelet disorders (40; 11%) and other rare bleeding disorders (9; 3%). Here, “treatment” refers to any medical or surgical intervention for managing HMB, including hormonal therapy or antifibrinolytic agents, as reported by participants in response to the question: “Have you received treatment for heavy menstrual bleeding (e.g., antifibrinolytic agents, hormonal therapy)?” This indicates that patients with more common or clinically recognized disorders were more likely to undergo HMB management.

**Table 9:** Chi-Square Test of Association Between Inherited Bleeding Disorder Type and Treatment Outcomes (N = 350) Data are presented as N (%). The chi-square test was used to assess the relationship between inherited bleeding disorder type and screening status frequency. Statistical significance was considered at p < 0.05*. A chi-square test assessed the relationship between bleeding disorder type and treatment outcomes. A significant association was found (χ²(3) = 8.869, p = 0.031), with patients diagnosed with von Willebrand disease and hemophilia more likely to have received treatment compared to other groups.

Inherited Bleeding Disorder Type	No	Yes	Total	χ² (df = 3)	p	Cramer’s V
von Willebrand disease	38 (11%)	99 (28%)	137 (39%)	-	-	-
Hemophilia	49 (14%)	77 (22%)	126 (36%)	-	-	-
Platelet disorders	35 (10%)	40 (11%)	75 (21%)	-	-	-
Other	3 (1%)	9 (3%)	12 (3%)	-	-	-
Total	125	225	350	8.869	0.031^*^	0.159

Table 10 indicates a strong relationship between age group and the identification of a bleeding disorder (χ²(3) = 88.76, p < 0.001, Cramer's V = 0.504). The proportion of diagnosed bleeding disorders was highest among participants aged 26-35 years (95; 27%) and 36-45 years (90; 26%), and lowest in the youngest group, aged 18-25 years (22; 6%). Older participants were more likely to report a confirmed diagnosis of a bleeding disorder. This age-related difference may reflect variations in healthcare-seeking behavior, prior medical events, or opportunities for diagnosis, rather than actual differences in disease prevalence.

**Table 10:** Association Between Age Group and Diagnosis of a Bleeding Disorder (N = 350) Data are presented as N (%). The chi-square test was used to assess the relationship between age group and the frequency of diagnosis of a bleeding disorder. Statistical significance was considered at p < 0.01**. Chi-square analysis was used to test the association between age group and diagnosis status. A strong relationship was found (χ²(3) = 88.76, p < 0.001), with diagnoses more frequent in women aged 26-45 years compared to younger participants.

Age	No	Yes	Total	χ² (df = 3)	p	Cramer’s V
18-25 years	68 (19%)	22 (6%)	90 (26%)	-	-	-
26-35 years	25 (7%)	95 (27%)	120 (34%)	-	-	-
36-45 years	20 (6%)	90 (26%)	110 (31%)	-	-	-
46-55 years	12 (3%)	18 (5%)	30 (9%)	-	-	-
Total	121	229	350	88.76	<0.001^**^	0.504

## Discussion

The study assessed the diagnostic value of the screening of IBDs in Pakistani women with frequent HMB. In our population, increased ISTH-BAT scores were significantly associated with the severity of menstrual bleeding on the MBQ. These findings are consistent with prior international studies, showing higher rates of menorrhagia and other gynecological complications in women with IBDs, and align with regional evidence from Pakistan, highlighting the prevalence and burden of HMB among women of reproductive age. This underscores the need for context-specific screening and awareness initiatives in Pakistani healthcare settings [2,15].

The ISTH-BAT scores were significantly higher in women with a family history of bleeding disorders, implicating a high bleeding tendency. These findings are corroborated by previous research, which found ISTH-BAT scores to be significantly higher in participants with suspected IBDs than in healthy control subjects, confirming the tool's usefulness in identifying familial bleeding risk [16]. We also found that women who had experienced a family history of bleeding disorders possessed significantly higher MBQ scores, signifying severe menstrual problems. This is consistent with existing evidence that there is a correlation between bleeding problems and heavy, painful menstruation, which tends to negatively impact overall quality of life [17].

We observed that participants who self-reported a previous diagnosis of a bleeding disorder had higher ISTH-BAT scores, indicating a greater tendency toward bleeding symptoms as captured by the questionnaire. This finding aligns with previous evidence on the description of the continuum of bleeding severity, where even mild bleeding disorders are associated with increased instances of bleeding manifestations compared to the general population [18]. Women with a diagnosed bleeding disorder had much higher scores on the MBQ in our research, showing more severe menstrual problems. This confirms previous evidence that women with bleeding disorders experience more frequent and severe HMB, which results in anemia and a low quality of life in most cases [4].

In our study, both ISTH-BAT and MBQ scores were higher in older women, indicating that older women experienced a higher bleeding tendency and faced more severe menstrual problems. This corresponds with prior evidence showing that heavy and abnormal menstrual bleeding increases with age predominance among women with IBDs, usually due to gynecological reasons and anovulatory menstrual cycles [19].

In our study, women with von Willebrand disease had the highest ISTH-BAT scores, as it has been demonstrated previously that bleeding scores are valid inputs in assessing objective, quantitative measures of bleeding severity in women with von Willebrand disease. These findings support the potential utility of standardized bleeding assessment instruments in distinguishing the bleeding burden among IBDs [20], and suggest that von Willebrand disease could be prioritized in screening and diagnostic protocols for women with chronic HMB in Pakistan, facilitating the timely identification and management of at-risk patients. Our MBQ analysis showed the most significant prevalence of severe menstrual bleeding in women with von Willebrand disease, consistent with previous evidence on the high prevalence of HMB in this population and its adverse effects on quality of life. This underlines the need for counseling and specifically regulated management of menstrual bleeding in women with von Willebrand disease [21].

Our results are similar to prior studies, which also showed that women with IBDs experienced significantly higher menstrual blood loss than controls. This confirms our finding that greater ISTH-BAT scores correlate strongly with greater MBQ scores [14]. We also found that older women had higher MBQ scores, consistent with the results of previous population-based studies, which also reported that HMB occurred more frequently in older women [22]. Additionally, we found that women who had a family history of bleeding disorders had more significant menstrual bleeding as well, which supports the notion that inherited hemostatic defects are related to greater menstrual problems and reduced quality of life [17].

Our findings demonstrated that women with a diagnosis of a bleeding disorder exhibited increased MBQ scores, and, as established in previous studies, bleeding disorders - especially von Willebrand disease - are associated with increased menstrual bleeding [4]. We also established that the nature of the bleeding condition produced differences in MBQ scores, which adds to earlier findings that women with diverse forms of bleeding disorders experience varying levels of menstrual and gynecological complications [23]. Finally, we found higher MBQ scores among women with other symptoms related to bleeding. These results align with previous findings, where females exhibiting other bleeding symptoms also reported elevated menstrual issues [24].

In our study, treatment outcomes varied widely among the different types of IBDs, with patients with von Willebrand disease and hemophilia demonstrating increased treatment needs. This observation aligns with other findings in the literature, which have shown that patients with von Willebrand disease and a higher bleeding index have a greater probability of receiving intensive care. This is a crucial factor to consider, as the kind of disorder, alongside its severity, influences clinical outcomes [25]. Our analysis revealed that the chances of developing a bleeding disorder were higher in older women, according to the existing data, which portrays an increased likelihood of diagnosis due to age-related changes and the gradual build-up of symptoms. This emphasizes the need for greater vigilance and consideration of bleeding disorders in older women [19].

Limitations and future directions

This study has several strengths, including a relatively large sample size, use of validated instruments (ISTH-BAT and MBQ) for standardized assessment, and a focus on an under-researched population of Pakistani women with chronic HMB. However, the research also has several limitations. First, it was based on a questionnaire-based evaluation process rather than a confirmatory laboratory process, which limits the capability to make categorical conclusions about IBDs. Second, selection bias could have been introduced by the convenience sampling of gynecology outpatient clinics in Islamabad and Rawalpindi, which constrains the generalizability of the findings to other areas of Pakistan, especially rural environments where access to care and awareness rates may vary. Third, the self-assessment of menstrual bleeding and symptoms associated with bleeding may be subject to recall and reporting biases, which could impact the validity of the severity measures. Also, some cultural taboos or stigma towards menstruation might have encouraged some participants to underreport symptoms. Lastly, the cross-sectional research design excludes the possibility of inference about causal associations among demographic-related variables, diagnosis status, and bleeding severity.

Future studies should combine standardized bleeding assessment tools with laboratory confirmation to improve diagnostic accuracy for IBDs. Conducting multicenter research that includes both urban and rural populations will provide a more representative understanding of the prevalence and burden of these disorders across Pakistan. Additionally, longitudinal studies are necessary to assess clinical outcomes, quality of life, and reproductive health following early diagnosis and effective management. Interventions to increase awareness among healthcare providers and patients, reduce diagnostic delays, and improve access to therapies for less common bleeding disorders should also be explored.

## Conclusions

The current study suggests that structured questionnaires may have potential value in identifying women at higher risk of hereditary bleeding disorders and chronic HMB in Pakistan. Female individuals who had family members with, or a confirmed diagnosis of, bleeding disorders had much higher bleeding scores as well as menstrual severity rates, which shows that early detection is clinically relevant. The results suggest the potential utility of introducing standardized screening tools in gynecological practice to identify women at risk of hereditary bleeding disorders - particularly younger women (18-25 years), who are often underdiagnosed - and to guide timely clinical assessment, potentially reducing complications such as iron deficiency anemia and unnecessary surgical interventions. To achieve better patient outcomes and improve quality of life, it is crucial to increase access to the diagnosis and management of IBDs, especially in regions where healthcare resources are limited.

## Appendices

**Table 11:** Demographic Information Questionnaire

Items	Responses
Age	-
Marital Status	-
Educational Level	-
Occupation	-
Do you have a family history of bleeding disorders (e.g., hemophilia, von Willebrand disease)?	-
Have you ever been diagnosed with a bleeding disorder?	-
If yes, which bleeding disorder were you diagnosed with?	-
Do you experience any of the following symptoms? Easy bruising, Frequent nosebleeds, Prolonged bleeding after cuts, Bleeding gums, Joint or muscle bleeding, None of the above	-
Have you received treatment for heavy menstrual bleeding (e.g., antifibrinolytic agents, hormonal therapy)?	-
Do you smoke?	-
Do you engage in regular physical exercise?	-

**Table 12:** International Society on Thrombosis and Haemostasis Bleeding Assessment Tool (ISTH-BAT) Data adapted from the ISTH-BAT, developed in 2010 by Rodeghiero et al. [13]. Permission to use the tool was obtained via email from the authors.

Items	Responses
Epistaxis?	-
Have you ever had spontaneous epistaxis?	-
Have the symptom ever required medical attention?	-
If answer to 1.2 is yes, please specify	-
How many times in your life did you receive any of the above treatments?	-
At what age did you first have symptoms?	-
Approximate number of episodes NOT requiring medical attention:	-
Duration of average single episode NOT requiring medical attention:	-
Cutaneous Bleeding (Bruising, Ecchymoses, Hematomas)	-
Have you ever had any of the above cutaneous bleeding?	-
Have the symptoms ever required medical attention?	-
If answer to 2.2 is yes, please specify (select all that apply):	-
How many times in your life did you receive any of the above treatments?	-
At what age did you first have symptoms?	-
Approximate number of episodes NOT requiring medical attention:	-
Type of bleeding:	-
Location of bleeding:	-
Common size of bleeding:	-
Bleeding from Minor Wounds	-
Have you ever had prolonged bleeding from minor wounds?	-
Have the symptoms ever required medical attention?	-
If answer to 3.2 is yes, please specify (select all that apply):	-
How many times in your life did you receive any of the above treatments?	-
At what age did you first have symptoms?	-
Approximate number of episodes NOT requiring medical attention:	-
Duration of average single episode	-
Menorrhagia (Heavy Menstrual Bleeding)	-
Have you ever had very heavy menstrual bleeding (menorrhagia)?	-
Have the symptoms ever required medical attention?	-
If answer is yes, please specify (select all that apply)	-
How many times in your life did you receive any of the above treatments?	-
At what age did you first have symptoms?	-
Have you had time off work/school due to menorrhagia?	-
Duration of menorrhagia	-
Have you had acute menorrhagia requiring emergency treatment/hospital admission?	-

**Table 13:** Menstrual Bleeding Questionnaire (MBQ) Data adapted from the Menstrual Bleeding Questionnaire (MBQ), developed in 2015 by Matteson et al. [14]. Permission to use the questionnaire was obtained through a licensing agreement (License Number 6096591257742).

Items	Responses
During the past month, how would you describe your periods?	-
On your heaviest day of bleeding during the past month, how many high absorbency sanitary products did you soak (either completely or almost completely)?	-
During the past month, how often did you need to wear either an incontinence brief or more than one high absorbency sanitary product (either more than one pad, a pad and a tampon, more than one tampon) at a time to contain your bleeding?	-
During the past month, how many times have you had an episode of bleeding that soaked through your “outer” clothes (pants, skirt, dress)?	-
During the past month, how many times did you need to get out of bed in the middle of night (or during sleep hours) to change your sanitary products?	-
During the past month, how many times did you pass blood clots (clumps of blood)?	-
During the past month, how often did passing blood clots (clumps of blood) stain your clothing?	-
During the past month, my period was associated with…	-
During the past month, how many weeks did your periods last?	-
During the past month, on how many days do you think your work at your job suffered because you were bleeding?	-
During the past month, on how many days did you miss work because you were bleeding?	-
During the past month, on how many days did you avoid family activities (grocery shopping, household chores) when you thought you would be bleeding?	-
During the past month, when would you carry sanitary products (pads, tampons) with you (in your pocket, in your bag)?	-
During the past month, on how many days did you avoid social activities (such as getting together with friends, going shopping for fun, going sight-seeing) when you thought you would be bleeding?	-
During the past month, on how many days did you plan your activities (work, social, or family) based on whether or not there was a bathroom nearby?	-
During the past month, on how many days did you bring extra clothes with you (to work, out shopping) in case you had staining from your period?	-
During the past month, on how many days did you choose what to wear based on whether or not you were bleeding?	-
On a scale of 0–10, with 0 being no concern at all and 10 being extremely concerned, please rate your overall concern about bleeding staining your clothes.	-
During the past month, would you say that your period start date was…	-
During the past month, would you say that your period end date was…	-
